# Collective movements of pedestrians: How we can learn from simple experiments with non-human (ant) crowds

**DOI:** 10.1371/journal.pone.0182913

**Published:** 2017-08-30

**Authors:** Zahra Shahhoseini, Majid Sarvi

**Affiliations:** Centre for Disaster Management and Public Safety, School of Engineering, The University of Melbourne, Australia; Waseda University, JAPAN

## Abstract

**Introduction:**

Understanding collective behavior of moving organisms and how interactions between individuals govern their collective motion has triggered a growing number of studies. Similarities have been observed between the scale-free behavioral aspects of various systems (i.e. groups of fish, ants, and mammals). Investigation of such connections between the collective motion of non-human organisms and that of humans however, has been relatively scarce. The problem demands for particular attention in the context of emergency escape motion for which innovative experimentation with panicking ants has been recently employed as a relatively inexpensive and non-invasive approach. However, little empirical evidence has been provided as to the relevance and reliability of this approach as a model of human behaviour.

**Methods:**

This study explores pioneer experiments of emergency escape to tackle this question and to connect two forms of experimental observations that investigate the collective movement at macroscopic level. A large number of experiments with human and panicking ants are conducted representing the escape behavior of these systems in crowded spaces. The experiments share similar architectural structures in which two streams of crowd flow merge with one another. Measures such as discharge flow rates and the probability distribution of passage headways are extracted and compared between the two systems.

**Findings:**

Our findings displayed an unexpected degree of similarity between the collective patterns emerged from both observation types, particularly based on aggregate measures. Experiments with ants and humans commonly indicated how significantly the efficiency of motion and the rate of discharge depend on the architectural design of the movement environment.

**Practical applications:**

Our findings contribute to the accumulation of evidence needed to identify the boarders of applicability of experimentation with crowds of non-human entities as models of human collective motion as well as the level of measurements (i.e. macroscopic or microscopic) and the type of contexts at which reliable inferences can be drawn. This particularly has implications in the context of experimenting evacuation behaviour for which recruiting human subjects may face ethical restrictions. The findings, at minimum, offer promise as to the potential benefit of piloting such experiments with non-human crowds, thereby forming better-informed hypotheses.

## Introduction

Understanding collective behavior of complex systems such as the flocking behavior of fish and birds[1] and ant colonies has intrigued a great deal of research attempting to understand unknown aspects of this phenomenon. A wide range of previous studies [2–6] indicates that regardless of the fact that collective pattern is an outcome of individuals, there appears to be similarities in governing rules and patterns emerged from different systems and groups with different shapes, sizes, objectives, and methods of communication. They believe despite the fact that each system enjoys its specific motion patterns, while a system constitutes of several interacting and similar members, universal behaviors (i.e. universal motion pattern) in which detailed nature of its component is being transcended are expected to be observed.

Growing interest towards an understanding of the collective behavior of non-human organisms as well as increasing number of studies that have been carried out to examine patterns of scale-free behaviour of pedestrian crowds under emergency conditions and under the impact of different architectures using biological entities inspired this study. In other words, in recent years, exploring the collective behavior of biological entities during extreme escape has received an increasing attention as an approach of gathering experimental evidence for exploring crowd dynamics under emergency conditions. This approach has been proved most useful for gaining basic insights as to aggregate measures of collective movements. Despite the fact that utilizing this innovative approach has received acceptability in the literature, the reliability of results obtained from this type of experiments remains to be discussed as to whether or not the findings emerged from these experiments, panicking non-human entities, would translate to human behavior in real context.

Moreover, modeling pedestrian crowd movement has received a growing recognition in the recent years due to an increase in crowd-related incidents around the world. In the spirit of guaranteeing the safety of pedestrians, an accurate understanding of pedestrian crowd behavior and the rules that govern their motion is of critical importance. As suggested by [7], the architecture of built environments especially the design of escape areas strongly influence on collective behaviour of crowds.Although some attempts of modeling and simulating pedestrian crowd motion[8–17] have been around, there has been very little knowledge as to the understanding of the impact of various built environments and geometrical features on crowd dynamics and collective motion of large number of people. The lack of knowledge mainly results from the difficulties involved in the provision of empirical data based on which different aspects of human crowd behavior can be scrutinized.

One of the methods of data provisioning that has been employed recently is experimenting with non-human entities particularly under extreme conditions of escape suggesting the practicality of this approach for gaining basic insights towards the collective patterns and characteristics of pedestrian flows under emergency conditions. Despite the great effort made to design and perform animal crowd experiments and despite the great deal of evidence that has emerged as a result, the literature does not offer sufficient evidence as to the extent of relevance of this experimental method. The method is completely non-invasive to humans (particularly for when the emergency escapes behaviour is of researchers’ interest which poses safety and ethical constraints for staging realistic experiments with humans) and is also often much less expensive compared to its equivalent human-experiment method. However, the literature lacks adequate evidence and systematic studies as to how reliably animal crowd models of behaviour can be used as a proxy for their human peers.

As Haghani and Sarvi [18] recently discussed in a review of all experimental methods in the field of crowd dynamics, the current literature demands systematic connections between animal and human experiments in order for us to have a better understanding of the extent of the relevance of animal crowd experiments. As discussed in their review, connections between experiments in realistic and virtual-reality environments has started to emerge [15, 19, 20] but similar connections between human and animal crowd experiments are yet to be established.

Up to now, mice and ants have been the most common organisms of experimentations. One of the early studies in this field [21] investigated the dynamic of groups of panicked mice escaping from a rectangular water container examining scale-free behavior and self-organized queuing. They pointed out that distribution of burst-size frequency for escaped mice shows (truncated) power-law and exponential distributions depending on the sampling rate and the width of exit. In another pioneer experiment, Altshuler, Ramos [22] studied groups of ants, as models of pedestrians, escaping from circular chambers with two symmetrically located exits to investigate the possibility of herding under different levels of panic conditions. Having changed the dose of repellent to create low and high-panic conditions, they observed symmetry breaking, non-symmetrical use of two identical exits, as a function of two controlling parameters; herding and panic parameter. Their findings suggest that emergence of symmetry breaking and follow-the-crowd behavior is more probable under high-panic conditions. Recently, Li et al[23] investigate the effect of density on symmetry breaking which has not been clearly taken into consideration in previous studies suggesting that increase in a total number of escaped ants decrease the degree of asymmetry.

A large number of studies using animal models of escape have explored the effect of architectural designs of the escape environment and their impact on evacuation efficiency. A group of studies of ant experiments [24–26] investigated the impact of the presence of column and obstruction near exits on the efficiency of the evacuation of groups of ants escaping from circular chambers. They found out that presence of column at exit improves the evacuation capability of chambers. In another related studies, this impact was also observed in sheep flocks [27, 28]. These studies reports on the impact of obstacle position on movement efficiency and points out that the distance of the obstacle to the exit can determine the positive or negative impact of presence of obstacle on emergency performance.

In order to understand the impact of conflicting geometries on the emergency response of pedestrian facilities, a number of studies [29, 30] investigated the effect of turning maneuvers on discharge efficiency of groups of panicked ants escaping from rectangular chambers which lead to an angled corridor (different angles). By comparing flow rates and velocity in angled corridors versus straight corridors, they found that the presence of turning angle greater than 45° can considerably decrease the flee times and output rate, particularly under high-density situations. another study[31] examined the effect of corridor configuration in merging passages using ant models of collective motion.

Another stream of studies has extensively practiced biological entities particularly panicked ants to gain insight towards complex phenomena such as ‘faster is slower’ effect which refers to a situation where elevated desire for escape may actually impede escape efficiency. There are studies that have addressed this particular topic with the use of animal experiments. In a study using ants panicked (repelled) by citronella escaping from triangular chambers with one narrow exit, Soria, Josens [32] observed a non-monotonic relation between the citronella concentration as a proxy of desire for flee suggesting the output rate of ants picking at certain intermediate levels and dropping afterward suggesting the emergence of FIS phenomenon. In other words, they showed that a minimum evacuation time was observed for an intermdiate level of cintronella concentration. In another study, Parisi, Soria [33] investigated jamming and clogging of panicked ants around exits escaping from a V-shaped chamber through a narrow exit and disputed that nature of observed FIS phenomenon in their previous study [32]. They attributed the observed slowness effect to an increase in frequentness of taking backward steps by ants. Using increased temperature as an alternative for creating panic conditions, Boari et al. [34] studied jamming and clogging and FIS phenomenon. They observed evacuation time decreases monotonically as a result of increasing the level of the aversive stimulus indicating FIF effect. Recently, experimental examination of this effect has been reported on the use of sheep [28, 35]. Using video records of sheep flocks passing through a narrow door, they [35] investigated the features of clogging regime and the impact of the presence of an obstacle at the exit. Their results suggest that the distribution of lapse times displayed a power law tail as well as the alleviation effect of the obstacle in preventing long flow interruptions. Using weather temperature as a proxy of competitiveness between animals exiting through the door, the authors [36] observed the emergence of FIS phenomenon with high competitiveness conditions gives rise to longer time lapses. Exploring this phenomenon, Lin, Ma [37] studied the escape time and burst size of groups of trained mice escaping through a narrow exit. Similarly, they reproduced the findings that rush level influences output rate suggesting “faster is slower” phenomenon. Yet, the majority of these studies have made no use of empirical data obtained from experiments with humans for validating their results against it.

Sobhani, Sarvi [38] examined the relation between flow rate and density at the exit of panicking woodlice escaped from rectangular chambers. They observed the output rate at the exit is variable, and that increasing discharge flow and density resulted in an increase in exit capacity. Using trained and untrained mice as the subject of experiments, another study [39] explored the impact of prior individual training on emergency response considering escape time as a proxy of the efficiency of evacuation. They observed trained mice exhibit more efficient evacuation while showing self-organized queuing. Also, they observed that untrained mice groups did not show queuing. In the most recent study of “symmetry breaking” phenomenon, Wang et al [40] studied the behavior of ants escaping from single exit chamber with the aim of investigating group formation. They observed that the size of exit affects the time interval between the passage of two consecutive ants as well as the average flee rate. However, their results do not suggest a linear relation between the mean flow rate and the width of exit. Afterward, in another study [41], they explored the impact of location of exits and spacing between them on escape efficiency via groups of panicking ants. Their findings illuminate emergence of symmetry breaking phenomenon undress stress conditions and no jamming at exits as well as the effect of spacing of two exits on egress efficiency (i.e. largest separation resulted in maximal evacuation efficiency). In another study[42], they investigated the relation between speed and density of ants escaping through a one direction passage. It was found that the relationship between components of traffic flow in humans and ants is not similar.

Based on this overview of the existing literature, there is limited knowledge regarding the consistency between conclusions drawn based on the experiments with non-human entities and what pedestrian crowds actually behave under emergency conditions. There is also limited evidence in the extent to which these conclusions can be generalized to the actual emergency conditions. Additionally, understanding the effect of geometrical features and design of merging streams on the motion patterns of human and non-human entities can be explored using experimental setups. As revealed through a comprehensive literature review, most of the previous studies in pedestrian crowd domain and biological domain have focused on investigating collective movements within simple environments and under normal condition (lower level of rush). However, there are limited studies which conducted to understand the effect of various architectural settings on the collective behavior.

## Materials and methods

In this study, we adopted two main approaches for gathering empirical evidence for examining the aforementioned issues in which humans and ants were the subjects of experiments. We investigate the emergency escape behaviour of pedestrians and ants evacuating crowded confined spaces. We quantitatively contrast the scale free behaviour of non-human entities along with collective movements of humans and estimated the distribution of output rate versus time, the probability distribution of time intervals between the passage of two successive entities and velocity distribution versus time and distance. The impact of different designs of merging configurations on the emergency response rate of escape areas is also investigated.

### Experiments with ants

**Fig 1:**
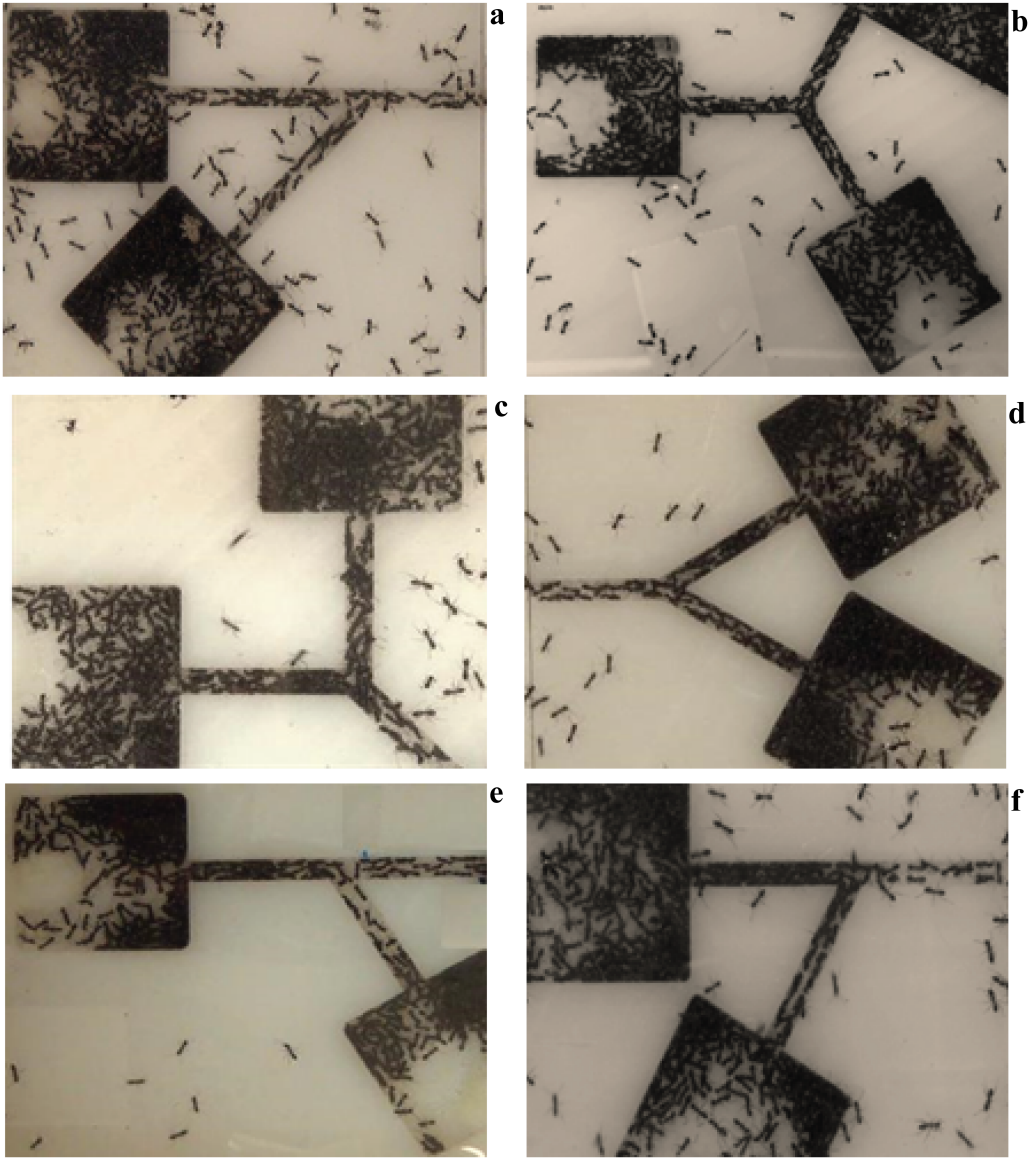
Snapshots of the merging experiments with panicking ants, (a) asymmetric 45° (b) symmetric 120° (c) Symmetric 90° (d) Symmetric 60° (e) Asymmetric 120° (f) Asymmetric 60°. The chambers are covered with transparent glasses. A certain dosage of repellant chemical is injected in each chamber, forcing the ants to quickly leave their nests. The two flows of ants have to merge with each other in a single passage in order to leave the environment.

Experimentation with non-human subjects allow a wide range of phenomenon to be explored that are otherwise hard to be replicated in experiments with human without concern about participants’ safety. In this study, we performed a wide range of experiments with Argentine ants (*Linepithema humile*), as abundantly available species in Melbourne. Further, according to Monash University Animal Ethics Office, animal ethics clearance is exempted for this particular species of ants (Monash University 2015). In these experiments desire to flee and speed are taken to extremes in order to observe how the patterns of movements of these organisms are influenced by the layouts of escape area and presence of conflicting maneuvers. In these experiments, the colonies of Argentina ants were collected from the various sites at the Clayton Campus of Monash University and prepared for experiments following the procedures describes in [43].In the laboratory experiments, six different designs of merging angle and configuration of paths were considered to explore the collective movements of panicking ants. The experimental designs are symmetric -60°, symmetric-90°, symmetric-120°, asymmetric-45°, asymmetric-60° and asymmetric-120° merging layouts. In symmetric layouts, two branches meet each other in a symmetrical way, and in asymmetrical setups, a straight branch is joined by another deviated stream. Fig 1 illustrates the snapshots of different merging layouts we examined. Each experimental setup is made up of two 25 mm by 25 mm chambers and contains an exit corridor of 3 mm width. The length of the corridor downstream and upstream of the merging point is 20mm. To minimize the probability of ants crossing on top of each other while they are escaping from chambers, the depth of the chambers is kept at approximately 3 mm. In addition, the chambers were covered with a transparent plastic lid, with a 1 mm diameter hole for injecting 10μl of citronella, to prevent ants escaping out from walls of corridors. Approximately 250 ants nest inside the chambers with a controlled level of humidness. The Citronella is inserted in cotton inside the chamber, through a hole in the cover of chambers, which spread outs the smell of Citronella and creates panic among ants. As a result, once the experiment starts, the number of ants near the exit areas dramatically increases which gives rise to the creation of saturated input flow rate in each branch of merging. Each experiment was repeated 12 times and they are recorded using a digital HD video camera and the recorded videos were visually examined. In addition, two dimensional velocity distributions were calculated converting those videos into image sequence. We examine the macroscopic features of movement measuring escape flow rate, flee time, speed and time interval between two successive ants at the downstream of the merging point.

### Experiment with human

**Fig 2:**
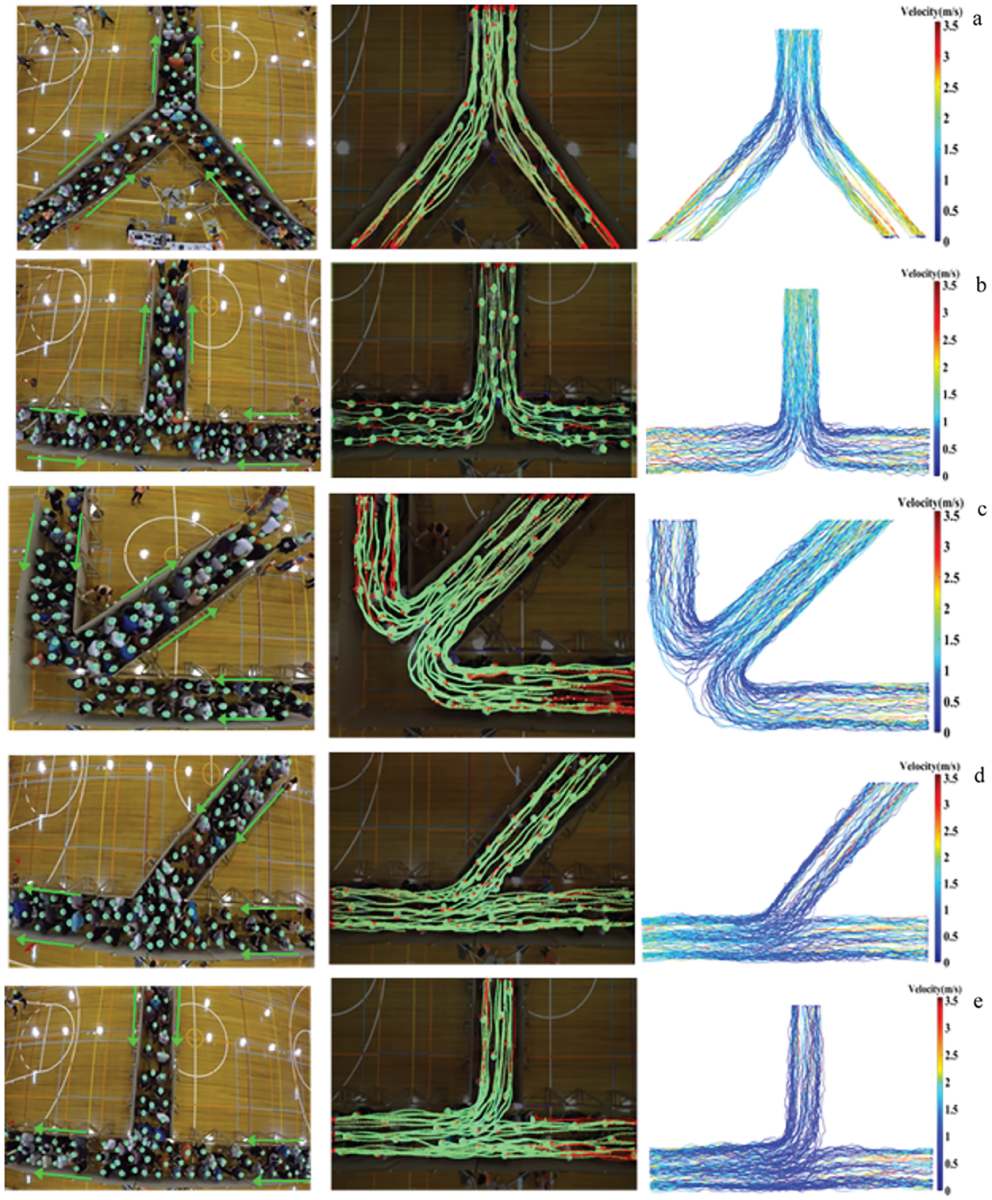
Snapshots from the raw footage of the experiments (on the left), the extracted trajectories appeared in the interface of the tracking software (in the middle) and the full trajectories of all subjects colour-coded based on their velocity along the corridor. (a) Symmetric 90° (b) symmetric 180° (c) symmetric 270° (d) asymmetric 45° (e) asymmetric 90°.

**Fig 3:**
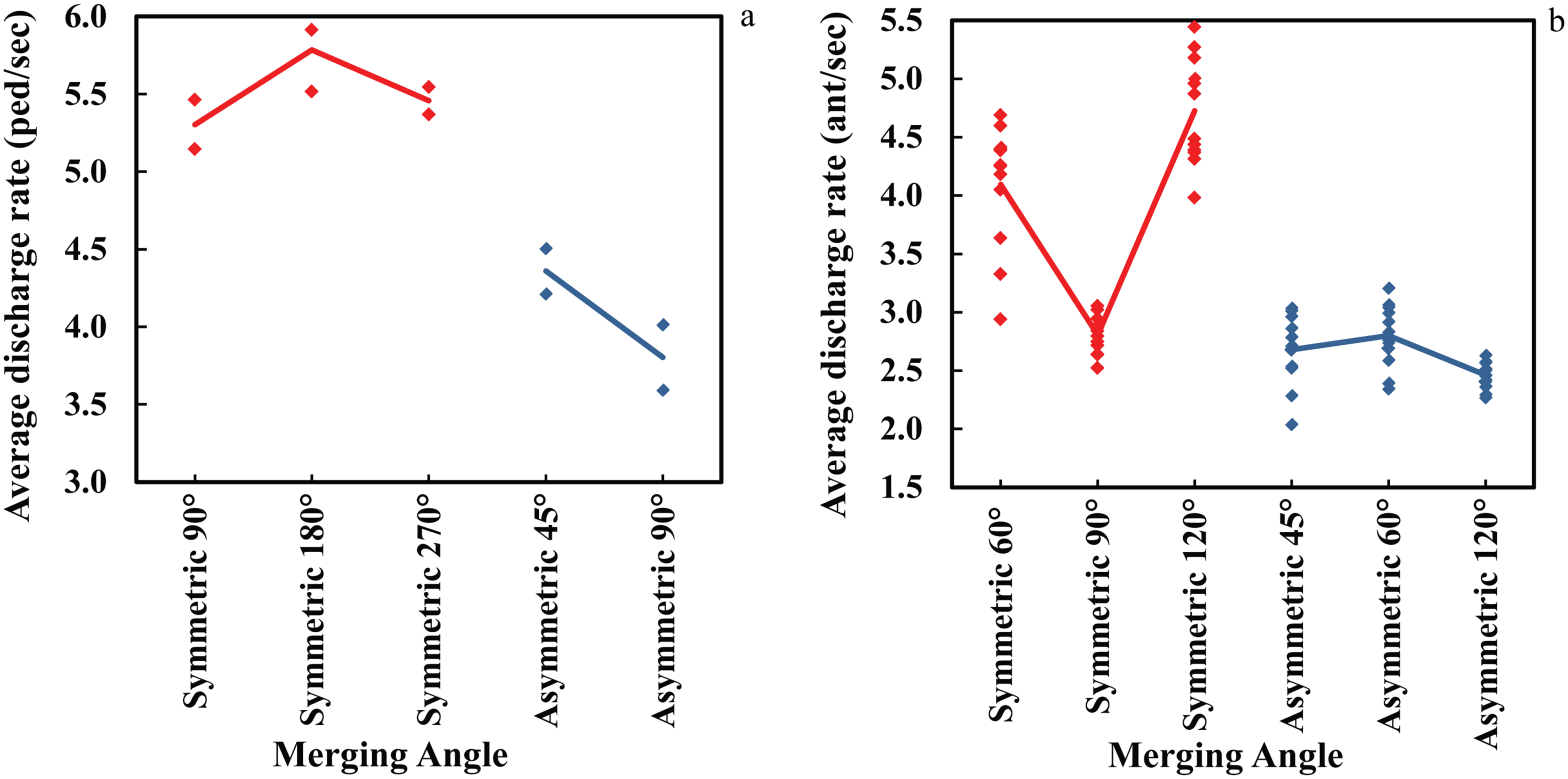
Comparison of average discharge rate among layouts of merging. (a) Experiment with human (b) Experiment with ants.

The experiments simulated emergency escape of human crowds in conflicting maneuvers. These simulated egress scenarios were conducted on 23th February 2015. The experimental procedure was approved by Monash University Human Research Ethics Committee and all the methods were carried out in accordance to the approval guidelines. Also informed consents were obtained from all subjects who accepted to participate in the experiments. We performed 30 evacuation scenarios in which factors such as merging angle, geometry, and desired speed level were varied. 150 participants were recruited and performed simulated emergency evacuation scenarios in a sport center in Melbourne, Australia. To minimize the possible effects of the testing on the participants, they were instructed to be mindful of others while competing with each other in performing the trial tasks at the beginning of the experiment. Also, in order to minimize the safety risks on the participants, soft and light wooden material was used for building the corridors. The geometric setups include symmetric-90°, symmetric-180°, symmetric-270°, asymmetric-45°, asymmetric-90° merging layouts. Fig 2 illustrates snapshots of the raw footage at these different scenarios. Through considering these different experimental setups, we intend to understand the importance of the design as well as the merging angle on the pedestrian crowd behavior. In these experiments, participants wore a green beanie with a centered black dot of a 4cm diameter which is used to mark their positions. At the beginning, they were held in waiting areas and when the experiment started, they moved through a 2-meter passage used as a buffer to minimize the effect of the entrance and having nearly homogenous flows over the entire width of corridors (1.5 meter). When they left corridors, they returned to the waiting area. Participants were asked to run and evacuate the experiments as fast as possible. Our participants had been asked to wear colorful beanies to facilitate the recognition of their positions. We extracted accurate movement trajectories of the subjects at each trial scenario for all individuals at the same time from recorded videos using a specialty software, Petrack [44] designed for the analysis of pedestrian’s movement trajectories in such experimental settings. The analysis software was accordingly set at the color recognition mode. The movement trajectory of each participant appeared in each trial run was extracted individually, while their head orientation and body movement being analyzed as they moved. Also, we calibrated the parameters in the software based on the conditions of our experiments. Fig 3 illustrates extracted trajectories and color trajectories. The color of trajectories in the second column represents pedestrians’ velocity along the flow direction.

## Results

### Escape rate analysis

In order to study the collective patterns of humans and ants while escaping as well as the impact of the existence of different merging configurations in the egress area, the average escape rates are calculated. The distribution of estimated average discharge rates observed in the experiments associated with each particular setup is presented in Fig 3.

According to the chart, there appears to be a remarkable level of agreement between the average discharge rate of pedestrian evacuees and escaped ants. The asymmetric merging setups deliver significantly poor performance in terms of average output rate compared to those in which the two flows meet each other symmetrically. Additionally, both figures suggest a dependency between the average discharge rate and the merging angle. Our findings confirm the significant role of the geometrical characteristics of merging layouts in terms of merging angle and design on the output rate. Both symmetrical and asymmetrical layouts exhibit sensitivity to the angle between merging branches. However, the relation between the average escape rate and the merging angle is suggested by both sets of observations to be non-monotonic. This suggests the importance of the design of merging areas and its impacts on evacuation response under emergency conditions.

There may be conceptual justifications for the performance differences displayed by different merging layouts. For instance, by comparing the movement patterns observed in the merging symmetric-180° and merging symmetric-90° (reflected in the trajectories presented in Fig 2), one can notice a more distinct pattern of motion self-organisation in symmetric-180° that may explain better discharge efficiency of this setup. As can be observed from Fig 2(b), pedestrians from two streams exhibit a great tendency to form and maintain stable lanes which is in accordance with observation from [45] suggesting that pedestrians from upstream-left corridor have a propensity to stay in the left side of downstream corridor and right side of shared pathway is mainly occupied by pedestrians from upstream-right corridor. However, in other merging setups, for instance asymmetric-45°, maintaining spatial segregation seems hardly practicable for participants. In other words, one can discuss that the setups in which the self-organisation patterns of pedestrian traffic are likelier to break down are also likelier to cause traffic instability and further delay in movement. This can be thought of as one possible explanation of the observed differences in the performance of different geometric layouts.

**Fig 4:**
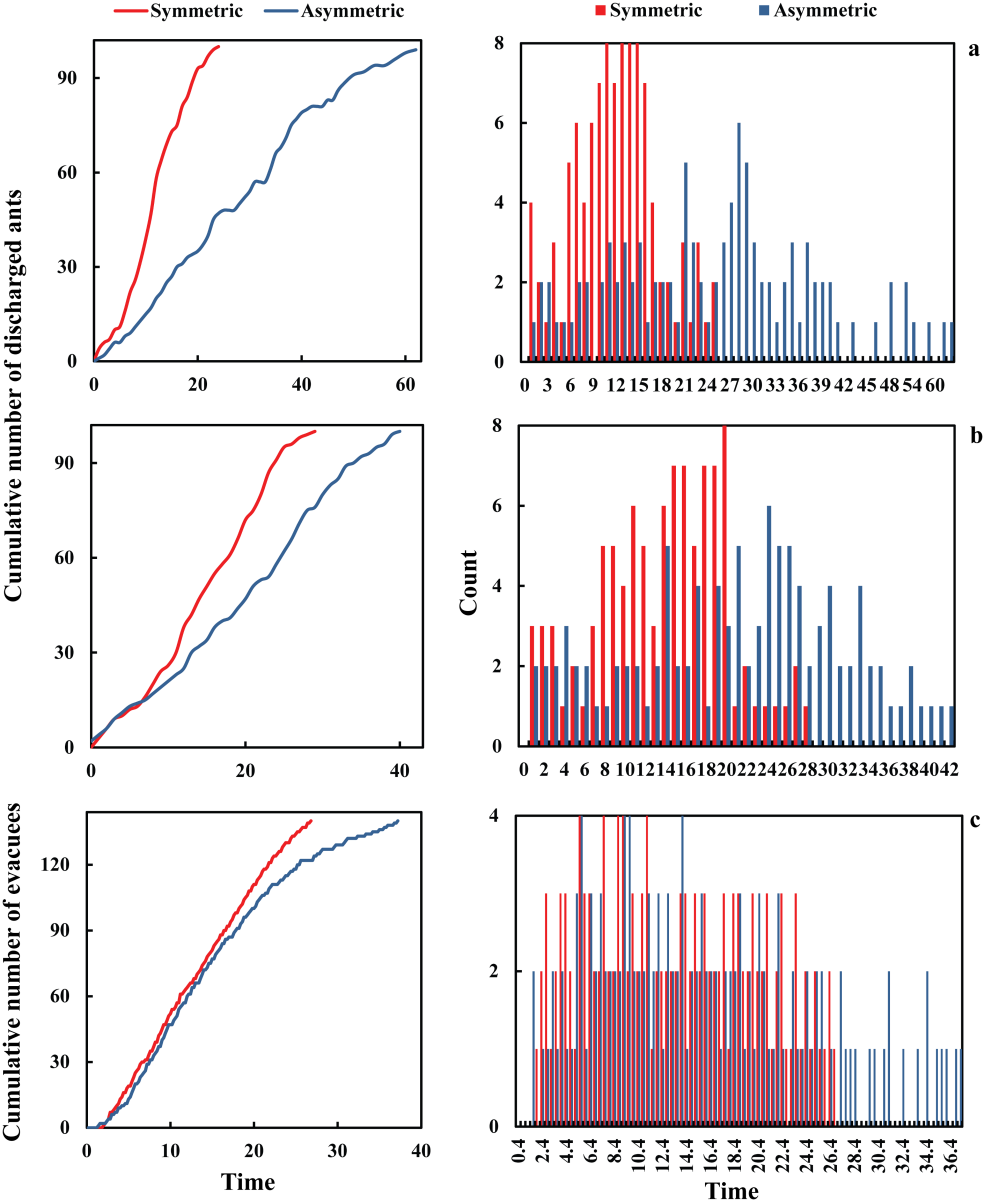
Comparison of discharge frequency between merging layouts with same angles and different configurations (i.e. symmetry/asymmetry). (a) Experiments with ants at merging angle = 120°, (b) Experiments with ants at merging angle = 60°, (c) Experiments with humans at merging angle = 90°.

In order to highlight the considerable difference between the performance of symmetric and asymmetric merging layouts, the cumulative number of evacuated pedestrians and escaped ants with respect to time has been depicted in Fig 4 for various designs of similar configuration as well as frequency distributions of discharged experimental subjects. It is shown that over each period of time, a merging setup in which two streams join symmetrically serve a significantly higher number of evacuees compared with a similar asymmetric configuration. Also, it is observable that asymmetrical merging layouts in both systems make the discharge flow more prone to be the intermittent compared to a similar symmetrical configuration. The nature of this intermittent behavior is observable in the figure where the horizontal and low-slope parts manifest the presence of clogging. This suggests the existence of asymmetrical merging layouts as an impediment of collective movements. These findings confirm the significant role of the design in terms of the way two streams meet each other supporting that a merging setup in which one stream need to impose itself to a straight stream might increase the likelihood incidents under emergency conditions due to serving a lower number of evacuees to exit.

### Headway distribution analysis

We explored the distribution of time lapse between the passage of two successive pedestrians and ants from the specific line at the downstream of different merging setups to quantitatively investigate the role of merging angle and design on the headway distribution.

**Fig 5:**
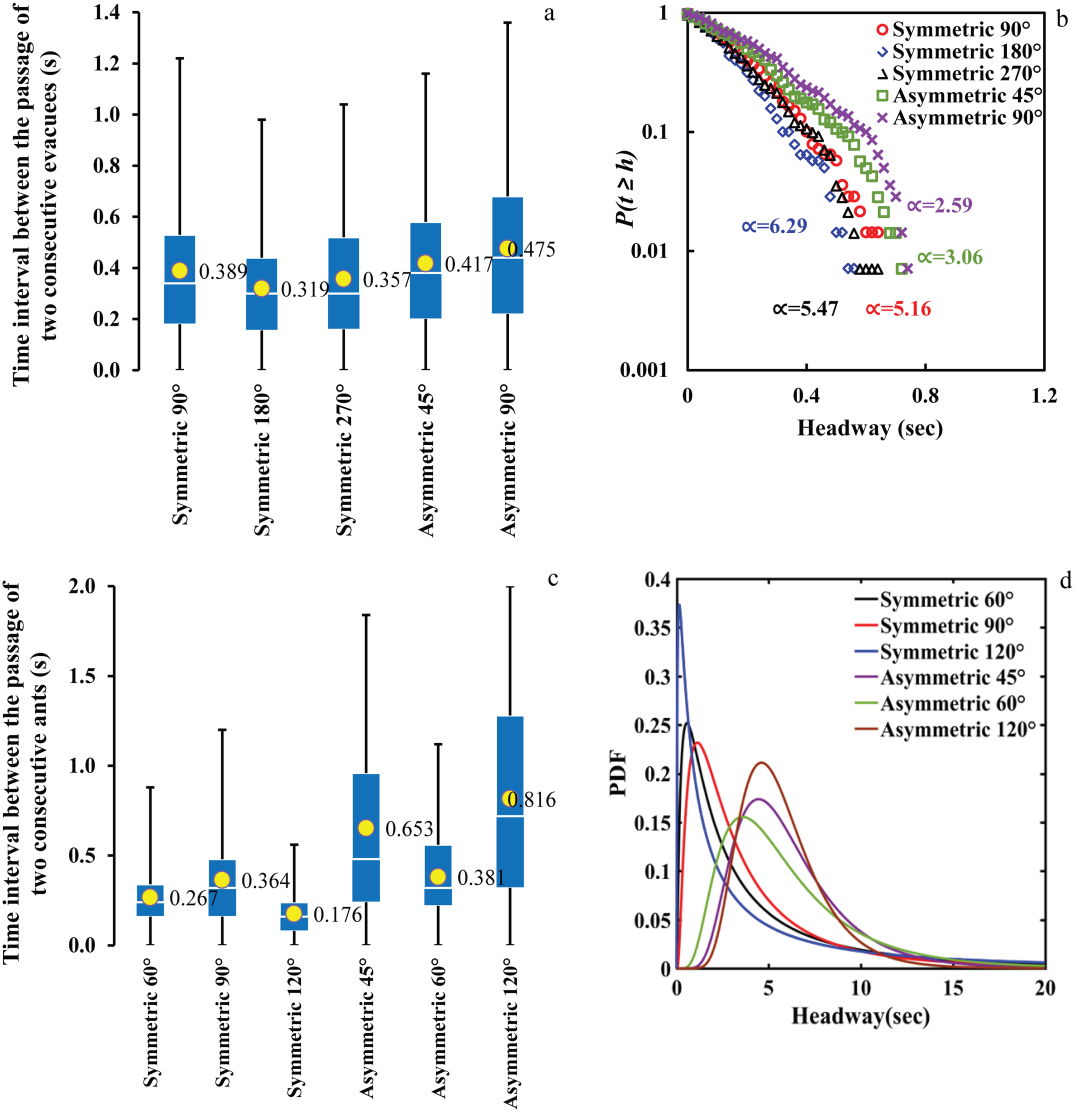
Comparison of headway distribution associated with different merging layouts. (a) Time interval between the passages of two successive participants-Human experiments (b) Time lapse complementary distribution function of headways obtained for the five different designs-Human experiment (c) Time interval between the passages of two successive ants- Ant experiment (d) headway distribution-Ant experiment.

The histograms of time lapse between the passages of two successive pedestrians suggest that the probability distribution of these time lapses exhibit a power-law tail with an exponent depends on the condition of the system in terms of the occurrences of clogging. This is in accordance with the findings of previous studies [28, 36, 46–48] in human evacuation. The complementary cumulative distribution of the time lapses associated with different merging layouts is demonstrated in Fig 5. Nonlinear fitting of the data associated with a histogram of headways between two consecutive ants shows that the probability distribution of the time intervals exhibits Galton distribution (Log-normal distribution). The fitted probability distributions of the headways are presented in Fig 5.

Comparing the headway distributions associated with different merging configurations highlight the fact that distribution of the time lapses exhibits a strong dependency on the features of the merging layouts (i.e. angle and design). In addition, analysis of human experiments reveals that complementary cumulative distribution function (CCDF) of pedestrian’s headway display slowly decaying tail for the asymmetrical merging setups and the corresponding power-law exponent is significantly lower for the asymmetrical merging settings. This suggests that the presence of asymmetrical merging configurations in escape area might increase the possibility of longer headways and blockage in both systems. According to the figure, in the system of panicking ants as well the curves corresponding to the symmetrical layouts are more skewed to the left indicating a shorter headway and less delay which is in consistence with the empirical evidence provided by our experiments with humans.

One can note the similarity between the patterns indicated by the left panel of Fig 5 (parts a and c) by measuring the average passage headway of each angle for ant and human experiments and their corresponding measurements of average discharge rate presented in Fig 3. The non-monotonic patter of performance change by the angle is observable based on both measurements as well as the distinct difference between the performance of symmetric and asymmetric layouts. The physical setups that are indicated best in terms of having the highest discharge rate also display smaller values of passage headways. The similarity of the findings based on these two measurements can also be used as evidence for further analysis given that they consistently led to by and large same conclusion in our case.

It is also noticeable (more distinctly based on plot 5(c) (ant experiment) and less distinctly based on plot 5(a) (human experiment) that the passage headway in downstream of the merging setup has greater variability in asymmetric setups than in symmetric setups as reflected in the length of the bow-and-whisker plots representing them. This could be a further indirect indication of more frequent traffic instability, flow interruption or transient clogging in such setups making quite large values of time intervals between successive pedestrians to be observable.

### Velocity analysis

**Fig 6:**
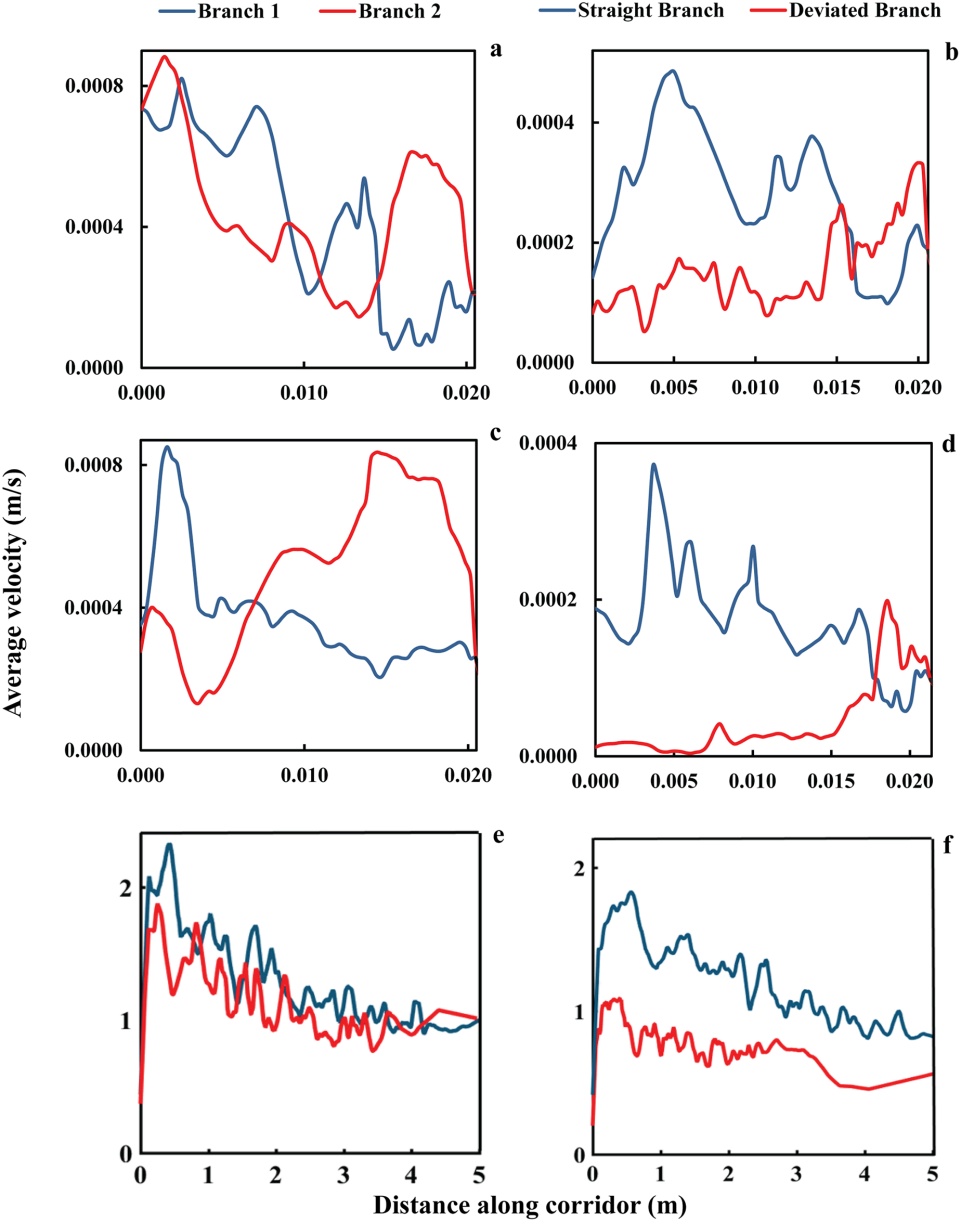
Spatial variations of velocities versus distance walked from the initial point of pedestrian (one end of corridor) measured along the corridor in which the pedestrians travelled. (a) Experiments with ants at merging angle and configuration = Symmetric 60° (b) Experiments with ants at merging angle and configuration = Asymmetric 60° (c) Experiments with ants at merging angle and configuration = Symmetric 120° (d) Experiments with ants at merging angle and configuration = Asymmetric 120° (e) Experiments with humans at merging angle and configuration = Symmetric 90° (f) Experiments with humans at merging angle and configuration = Asymmetric 90°.

To investigate the impact of merging configurations in escape areas on the collective movement of evacuees in terms of distribution of speed, average escape velocities along the corridors of the experimental layouts were estimated. We used a particle image velocimetry tool PIVLAB [49] for the system of panicking ants and extracted trajectories for the human experiments. Emphasizing on the impact of merging design regardless the absolute value of the angle between the two merging streams, the average spatial variation of the velocities in merging branches of similar merging configurations is visualized in Fig 6.

The speed analysis reveals the formation of “stop and go” phenomenon in the flow of the merging branches which causes the flow in one of the merging streams stop or experience a very low speed compared to the other stream. This crowd turbulence phenomenon has been reported by [50]. As can be seen from the figure, it is strongly suggested by both sets of observations that, the imbalance between the velocities of the two merging streams is observable in both symmetric and asymmetric setups. However, the imbalance between the velocities of single branches is much more severe in the asymmetrical merging settings. This means the creation of clogging is more observable in the deviated branch of the asymmetrical merging configurations.

**Fig 7:**
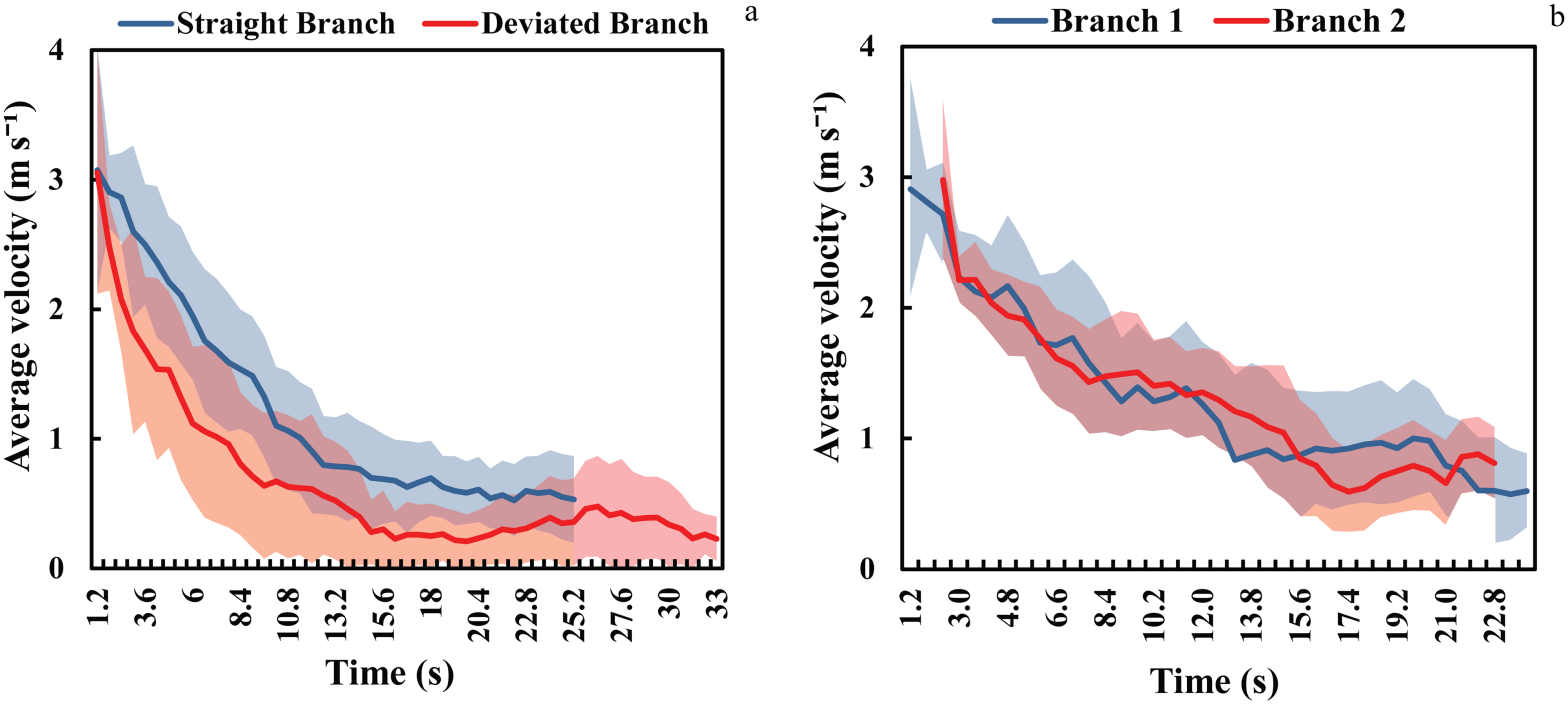
Temporal variations of velocities (a) asymmetric 90° (b) symmetric 90°.

Temporal fluctuations of the velocities in two single branches for human experiments under similar merging configurations are illustrated in Fig 7. According to this figure, the imbalance between the speed of the two merging branches might contribute to the creation of periodic clogging at upstream of a merging point. The temporal variations emphasize the important role of the design of merging layouts in relation to the balance between the speeds of the two merging streams. It can be observed from the estimated speed distributions that in symmetric design, the speed of two branches remains relatively close to each other. However, in the asymmetric design, speed in the straight branch is greater almost at all times. This means much longer evacuation time for the deviated branch and unbalanced flow at an asymmetric merging setup.

**Fig 8:**
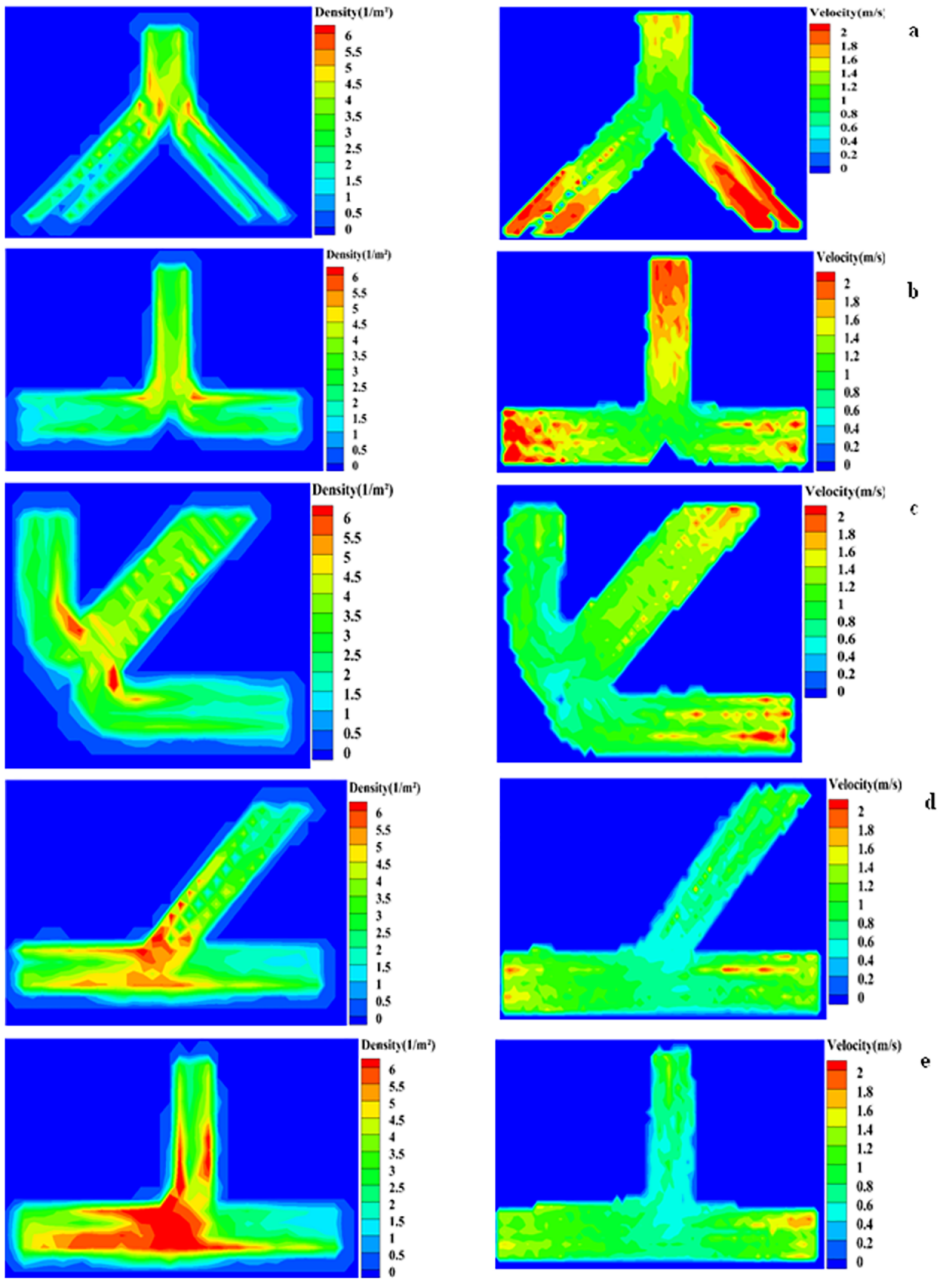
Color-coding the temporal average of density and the spatio-temporal average of velocity in experiments with humans. (a) Symmetric 90° (b) Symmetric 180° (c) Symmetric 270° (d) Asymmetric 45° (e) Asymmetric 90°.

Using the trajectories information we also measured the density and average velocity of the subjects at each point in time and space through discretisation of the evacuation space as well as the evacuation time. The calculated spatial averages of velocities and densities were also averaged over time for each scenario and visualised using colour coding methods in order to understand the considerable difference in velocity and density variations and fluctuations associated with the single branches in different merging layouts. Fig 8 provides details of colour-coded graphs velocity and destiny for each trial run. Also, see S3 to S7 Videos for dynamic videos that respectively colour-code the spatiotemporal averages of velocities and densities during each experiment scenario at a temporal resolution of 0.2 second.

Detailed analysis of velocity and density reveals that distribution of densities is not homogeneous and the density near the junctions is higher than other parts of the merging setups resulting in a abrupt reduction of velocity in those areas. In addition average density in the area that two single branches start to join each other in asymmetrical merging configurations is severely high leading to considerably low velocity in this type of merging setups.

## Conclusions and discussion

Whether there is any element of truth to be learnt from such experiments and the type of questions that can be addressed using animal crowd movement experiments as models of their human counterparts have been largely unknown requires replication of crowd motion experiments in both contexts. Systematic links between the contexts of the collective movement of humans and non-humans has so far been scarce in the literature. Given the non-invasiveness and relative logistical easiness of experiments with non-human entities as opposed to staging similar experiments with large groups of people, the answer to this question could have significant implications for the continuation of research in the field of crowd dynamics. Here in this work, we reported on linking between collective motion of humans and ants in experimental conditions as an attempt to bridge this gap.

Based on previous studies, some degree of similarity between the collective motions of different biological systems has been observed. However, it is highly unknown that whether or not inferences drawn based on experiments with non-human organisms are consistent with the actual behavior of pedestrian crowds in near field laboratory experiments or to what extent they are deviated from the real human behaviour. In other words, the extent to which and the circumstances and contexts under which findings generated from experimentation with non-human organisms can be relied upon as the proxy of the actual behaviour of pedestrian crowds in natural settings remains to be investigated.

In this study, a large number of experiments are conducted with panicking ants and humans providing the possibility of observation and quantitative analysis of the collective movements. A comprehensive data analysis was conducted by examining the motion trajectory of the participants which provides some measures of their scale-free behaviour. This led to findings both in terms of crowd behavior during evacuation from conflicting geometries as well as findings related to degree of resemblance between the collective pattern of movements emerged from both data sets. In other words, the laboratory experiments with human also shed interesting insights into the dynamics of human crowds during escape from environments that impose conflicting maneuvers (i.e. merging corridors).

The results inferred from the collective movements of humans under emergency condition (participants were asked to run and evacuate the setting as quick as possible) were in some way the same as those obtained from the experiments with non-human entities. This suggests that extreme emergency conditions might lead to the emergence of scale-free behavior. With regard to the resemblance between the collective movements of humans and ants, our results confirm that the patterns associated with the velocity variations and the fluctuations derived from both set of observations shares a remarkable degree of similarity in terms of the creation of stop-and-go phenomenon and severe imbalance between the distribution of velocities of the two merging branches over space and time. In addition the analysis of the escape rate in both systems suggest that despite the considerable dependency between the average escape rate and its distribution over time and merging angle, the relation between them does not prove to be monotonic.

However, there are certain limitations that need to be taken into consideration in terms of drawing possible generalisation to all escape concepts since this single work cannot be regarded as adequate proof for the reliability of ant (or other animal) crowd experiments, although it offered promising evidence to their possible relevance at least at the level of collective motion and based on aggregate measures. Much further evidence, however, is required for a better understanding of the possible similarities between the dynamics of humans and non-human crowd traffic. We believe data collected from experimentation with two organisms as complements suggesting more of experiments with human is required to make solid conclusions as to the limits of generalizability and applicability of experiments with non-human entities.

## Supporting information

S1 Video Raw video footage of scenario symmetric-180°. (WMV)

S2 Video Raw video footage of scenario asymmetric-45°. (WMV)

S3 Video Dynamic colour-coding of spatiotemporal averages of velocities in scenario symmetric-90° with a time resolution of 0.2 sec. (AVI)

S4 Video Dynamic colour-coding of spatiotemporal averages of velocities in scenario symmetric-180° with a time resolution of 0.2 sec. (AVI)

S5 Video Dynamic colour-coding of spatiotemporal averages of velocities in scenario symmetric-270° with a time resolution of 0.2 sec. (AVI)

S6 Video Dynamic colour-coding of spatiotemporal averages of velocities in scenario asymmetric-45° with a time resolution of 0.2 sec. (AVI)

S7 Video Dynamic colour-coding of spatiotemporal averages of velocities in scenario asymmetric-90° with a time resolution of 0.2 sec. (AVI)

S8 Video Dynamic colour-coding of densities in scenario symmetric-90° with a time resolution of 0.2 sec. (AVI)

S9 Video Dynamic colour-coding of densities in scenario symmetric-180° with a time resolution of 0.2 sec. (AVI)

S10 Video Dynamic colour-coding of densities in scenario symmetric-270° with a time resolution of 0.2 sec. (AVI)

S11 Video Dynamic colour-coding of densities in scenario asymmetric-45° with a time resolution of 0.2 sec. (AVI)

S12 Video Dynamic colour-coding of densities in scenario asymmetric-90° with a time resolution of 0.2 sec. (AVI)

S1 File Extracted movement trajectory. (7Z)
